# Foxp3 + Treg-derived IL-10 promotes colorectal cancer-derived lung metastasis

**DOI:** 10.1038/s41598-024-80437-8

**Published:** 2024-12-16

**Authors:** Ahmad Mustafa Shiri, Mohammad Fard-Aghaie, Tanja Bedke, Eleftherios D. Papazoglou, Morsal Sabihi, Dmitra E. Zazara, Siwen Zhang, Jöran Lücke, Philipp Seeger, Maximilian Evers, Thilo Hackert, Karl J. Oldhafer, Gabriel E. Gondolesi, Samuel Huber, Anastasios D. Giannou

**Affiliations:** 1 Section of Molecular Immunology and Gastroenterology, I. Department of Medicine, University Medical Center Hamburg-Eppendorf, 20246 Hamburg, Germany; 2 Hamburg Center for Translational Immunology (HCTI), University Medical Center Hamburg-Eppendorf, 20246 Hamburg, Germany; 3 Department of General, Visceral and Thoracic Surgery, University Medical Center Hamburg-Eppendorf, 20246 Hamburg, Germany; 4 Division for Experimental Feto-Maternal Medicine, Department of Obstetrics and Fetal Medicine, University Medical Center Hamburg-Eppendorf, Hamburg, Germany; 5 University Children’s Hospital, University Medical Center Hamburg-Eppendorf, Hamburg, Germany; 6 Division of Hepatobiliary and Pancreatic Surgery, Department of Surgery, Asklepios Hospital Barmbek, Hamburg, Germany; 7 Semmelweis University Budapest, Asklepios Campus Hamburg, Hamburg, Germany; 8 General Surgery, Liver, Pancreas and Intestinal Transplantat Unit, Hospital Universitario-Fundación Favaloro, Buenos Aires, Argentina; 9 Section of Molecular Immunology and Gastroenterology, I. Department of Medicine, Center of Internal Medicine and Department of General, Visceral and Thoracic Surgery, University Medical Center Hamburg-Eppendorf, 20246 Hamburg, Germany

**Keywords:** Immunology, Interleukins

## Abstract

The lung is one of the most frequently metastasized organs from various cancer entities, especially colorectal cancer (CRC). The occurrence of lung metastasis correlates with worse prognosis in CRC patients. Here, we aimed to investigate the role of IL-10 in lung metastasis development and identify the cellular source and target cells of IL-10 during lung metastatic establishment. To induce lung metastasis in mice, we injected MC38 murine colon cancer cells intravenously. Mice with *Il10*-deficiency were used to test the role of IL-10. The lung metastatic burden was assessed both macroscopically and histologically. IL-10- and Foxp3-reporter mice were employed to identify the cellular source and target cells of IL-10 in lung metastasis using flow cytometry. These findings were further confirmed using mice with cell-specific deletion of *Il10*- and IL-10 receptor (*Il10ra*). Interestingly, *Il10* ablation led to reduced lung metastasis formation, suggesting a pathogenic role of IL-10 in lung metastasis. Moreover, using reporter mice, we identified Foxp3 + regulatory T cells (Tregs) as the predominant cellular source of IL-10 in lung metastasis. Accordingly, Foxp3 + Treg-specific deletion of *Il10* resulted in decreased lung metastasis formation. In terms of target cells, myeloid cells and Foxp3 + Tregs expressed high IL-10Ra levels. Indeed, IL-10 signaling blockade in these two immune cell populations resulted in reduced lung metastatic burden. In conclusion, Foxp3 + Treg-derived IL-10 was found to act on Foxp3 + Tregs and myeloid cells, thereby promoting lung metastasis formation. These findings provide insights into lung metastasis-related immunity and establish the groundwork for optimizing metastasis-targeting immunotherapies through targeting of IL-10 as a novel therapeutic strategy.

## Introduction

Colorectal cancer (CRC) remains one of the most prevalent and deadly forms of cancer worldwide, with high incidence of distant metastasis, which leads to worse prognosis^1,2^. Lung metastasis in CRC represents a complex process that involves the migration of cancer cells from the primary tumor site to the lungs. This migration occurs through a series of steps known as the metastatic cascade, comprising local invasion, intravasation, survival in the circulatory system, extravasation, and colonization in the pulmonary environment^3^. Since the lung is one of the most frequently targeted organs, lung metastasis significantly contributes to the mortality rate associated with CRC^4^. Therefore, understanding how colorectal cancer cells establish secondary colonization in the lung is crucial for developing targeted therapies that improve patient outcomes.

Recent studies have highlighted the importance of immune system interactions in various cancer entities^5–8^. Previously, we have identified Interleukin-10 (IL-10) as a critical cytokine in the process of CRC-derived liver metastasis formation^9^. Specifically, Foxp3 + regulatory T cell (Treg)-derived IL-10 acted on monocytes and promoted liver metastasis formation via the upregulation of programmed death-ligand 1 (PD-L1)^9^. However, the role of IL-10 in lung metastasis has not yet been fully understood. IL-10 is well known for its anti-inflammatory and immunosuppressive capabilities, affecting a wide range of diseases^10–12^. IL-10 binds to the receptor IL-10R, majorly IL-10Ra, to initiate a cascade of signaling activation that broadly modulates the immune response^13,14^. The expression of IL-10Ra is relatively narrowly distributed, primarily found on cells of hematopoietic origin, including certain subsets, such as T cells, B cells, macrophages, and dendritic cells (DCs)^10,15^. This selective expression allows for a better definition of cells responding directly to IL-10.

IL-10 is a pleiotropic cytokine and has divergent roles in different diseases^13,16^. The effects of IL-10 vary according to the experimental setting and the cell types of interest. In metastasis, the traits of disseminated cancer cells show organ-specific patterns^17,18^. It warrants clarification whether IL-10 shares the same role in different metastatic sites, and more interestingly, whether the cellular source(s) and target cell(s) of IL-10 in different metastatic sites are the same. This is of high interest in clinical practice, since side effects upon systemic treatment are ought to be considered beforehand. For example, if the role of IL-10 is paradoxical in lung and liver metastasis, IL-10 inhibition can reduce liver metastasis, while adversely, facilitating lung metastasis. Therefore, more caution should be taken when employing systemic IL-10 treatment. Addressing this could ameliorate the management of colorectal cancer patients with distant metastasis by inhibiting IL-10 signaling systemically, or in an organ-specific way.

In this study, we aimed to investigate the role of IL-10 in lung metastasis using mouse models. In addition, we aimed to identify the major cellular source and target cells of IL-10 during lung metastasis. Taken together, our findings seek to provide novel insights into lung metastasis formation and uncover novel immunotherapeutic targets, such as IL-10, thereby optimizing treatment approaches for CRC patients with distant metastasis.

## Materials and methods

### Mice

*C57BL/6 J, Il10*^*-/-*^*, Il10*^*flox/flox*^*;Foxp3*^*cre*+^ *, Il10*^*eGFP*^*;Foxp3*^*RFP*^*, Il10ra*^*flox/flox*^*;Lysm*^*cre*+^ *, Il10ra*^*flox/flox*^*;Foxp3*^*cre*+^ mice were housed in the animal facility of the University Medical Center Hamburg-Eppendorf under specific pathogen-free conditions. *Il10ra*^*flox/flox*^ mice were kindly provided by Prof. Richard Flavell. The *Il10*^*flox/flox*^ and *Il10ra*^*flox/flox*^ mice have previously been validated and characterized^12,19^. Age- (8–14 weeks) and sex-matched littermates were used for experiments. All animal experiments were approved by the Institutional Review Board “Behörde für Justiz und Verbraucherschutz, Lebensmittelsicherheit und Veterinärwesen” (Hamburg, Germany). All methods were performed in accordance with the relevant guidelines and regulations. The study is reported in accordance with ARRIVE guidelines (https://arriveguidelines.org).

### Cancer cell lines

Colon adenocarcinoma (MC38) cancer cells were cultured in 10% FBS DMEM medium with penicillin–streptomycin. Cells were cultured in 37 °C incubator under 5% CO_2_. The cells were split using trypsin–EDTA (0.25%) once they had reached around 80% confluency. Similarly, cells with around 80% confluency were harvested for lung metastasis induction.

### Mouse models for lung metastasis induction

Forced lung metastasis was induced by injecting cancer cells intravenously (i.v.)^20^. As described previously, 100 μL of the resuspended cancer cells were administered into the tail vein of mice under anesthesia. The lungs were harvested 3 weeks post injection for lung metastatic burden assessment.

### Immune cell isolation

Murine lungs were harvested after PBS perfusion through the vena cava. Subsequently, the lungs were cut into small pieces and digested in a shaking incubator at 37 °C for 25 min. The media used for digestion of the organs consisted of HBSS (with Ca^2+^ and Mg^2+^) containing 10 U/ml DNase and 1 mg/ml Collagenase. After digestion, the lungs were smashed using metal cell strainers and washed using 1% FBS PBS into a 50 ml falcon. After centrifugation at 400 g for 8 min, the pellet was collected. Immune cells were then enriched from the pellet by Percoll gradient centrifugation (GE Healthcare, Chicago, IL).

### Flow cytometry

Lung immune cells were isolated as mentioned above. To block the Fc-γ receptors, a mAb (clone 2.4G2) was used. The cells were then washed and stained with fluorochrome-conjugated antibodies (Supplementary Table 1) for 15 min at 4 °C. The BD LSRFortessa (BD Biosciences, San Jose, CA) was used for flow cytometry. Data analysis was performed using FlowJo v.10 (TreeStar, Ashland, OR).

### Hematoxylin and Eosin (H&E) staining

Lung specimens were immediately fixed after harvesting in 4% buffered formalin. Subsequently, lungs were embedded in paraffin or OCT embedding matrix (Sakura, Tokyo, Japan) and stored at room temperature or -80 °C, respectively, for future staining. Before staining, lung tissue sections (4 mm) were prepared and stained with H&E. Pictures were taken using the Axio Vert.A1 (Zeiss, Jena, Germany) microscope.

### Statistical analysis

All data were analyzed using the GraphPad Prism statistical software (GraphPad software, San Diego, CA, USA). Mouse data are presented as mean ± SEM. Comparison of means was performed using the Mann–Whitney *U* test for paired group comparisons or the one-way ANOVA (Bonferroni) for multiple group comparisons, as appropriate. *P*-values below 0.05 were considered significant.

## Results

### Il10-deficiency protects mice against CRC-derived lung metastasis

**Fig. 1:**
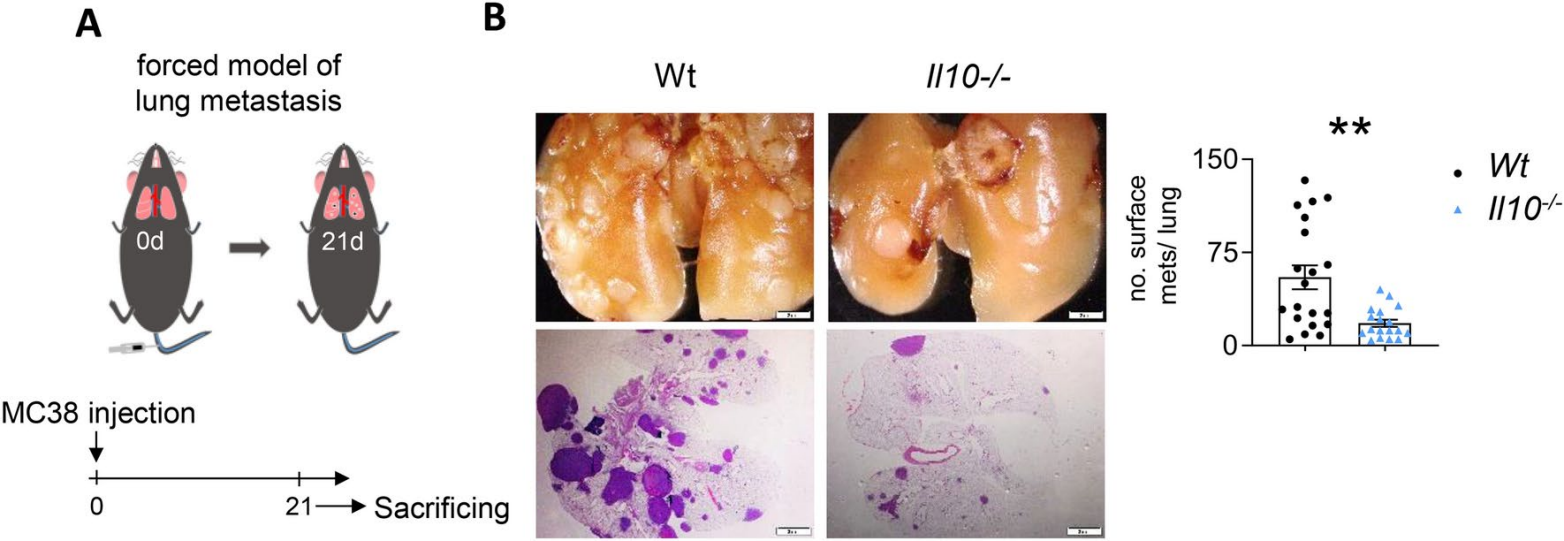
*Il10*-deficiency protects mice against CRC-derived lung metastasis. (**A**) Schematic overview of forced lung metastasis induction following intravenous MC38 cancer cell injection. (**B**) Representative macroscopic and histological pictures of lung metastasis following H&E staining, as well as number of macroscopic lung metastases in Wt and *Il10-/-* mice (n ≥ 18 mice per group). Scale bar: 2 mm. Data are presented as mean ± SEM. **p ≤ 0.01calculated by Mann–Whitney *U* test.

To pinpoint the role of IL-10 in CRC-derived lung metastasis formation, we injected MC38 colon cancer cells i.v. in *Il10-/-* and wild type (Wt) mice, and assessed the lung metastatic burden 21 days later (Fig. 1A). Interestingly, *Il10-/-* mice developed significantly fewer lung metastatic sites compared to the Wt mice (Fig. 1B), thereby suggesting a pathogenic role of IL-10 during CRC-derived lung metastasis formation.

### Foxp3+ Tregs are the major source of IL-10 during lung metastasis formation

**Fig. 2:**
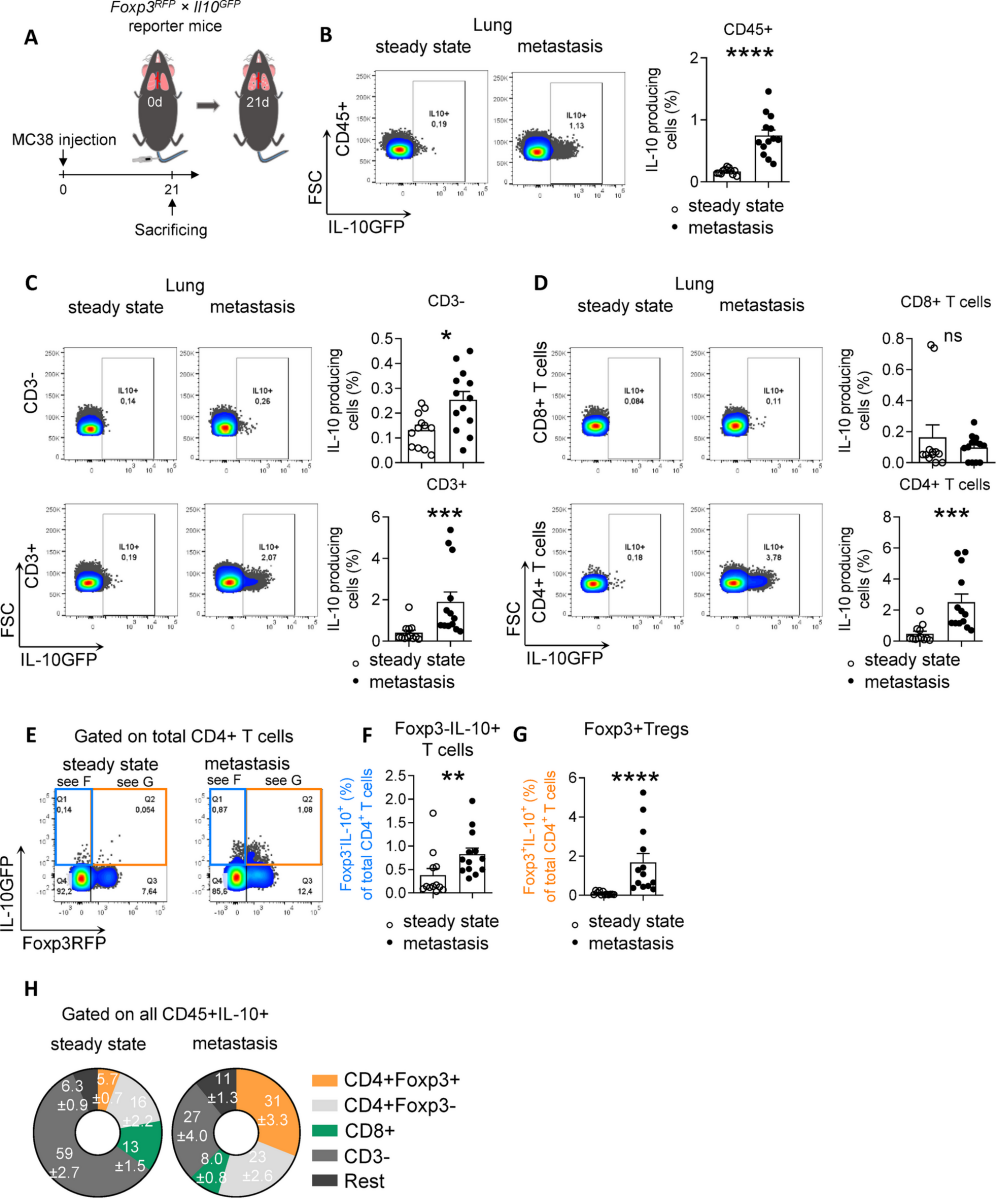
Foxp3 + Tregs are the major source of IL-10 in lung metastasis formation. (**A**) Schematic overview of the forced lung metastasis induction using intravenous injection of MC38 cancer cells in *Foxp3*^*RFP*^; *Il10*^*GFP*^ reporter mice (n ≥ 12 mice per group). (**B**–**D**) Frequency of IL-10 + cells in (**B**) all CD45 + cells, (**C**) CD3- cells and T cells, and in (**D**) CD8 + T cells as well as in CD4 + T cells. (**E**–**G**) IL-10 expression in (**F**) Foxp3- IL-10 + cells and (**G**) Foxp3 + Tregs. (**H**) General distribution of all IL-10 producing CD45 + cells in healthy lung and lung with metastasis. Data are presented as mean ± SEM. Non-significant (ns): p > 0.05; *p < 0.05; **p ≤ 0.01; ***p < 0.001, as calculated by Mann–Whitney *U* test.

To better understand how IL-10 signaling affects lung metastasis formation, we next aimed to identify the cellular source of IL-10 during lung metastasis formation. We have previously identified Foxp3 + Tregs as the major producer of IL-10 during liver metastasis formation^9^. To investigate whether Foxp3 + Tregs are the predominant cellular source of IL-10 in lung metastasis as well, a *Il10*^*GFP*^; *Foxp3*^*RFP*^ reporter mouse was used for lung metastasis induction (Fig. 2A)^21^. Compared to steady state, IL-10 production in lung metastasis increased significantly (Fig. 2B). Of note, increased IL-10 production was not only seen in CD3- immune cells, but also in T cells, especially in CD4 + T cells (Fig. 2C,D). Specifically, increased IL-10 production was found both in Foxp3 + Tregs and in Foxp3-CD4 + T cells (Fig. 2E–G). To pinpoint the population that contributed the most to IL-10 production in lung metastasis, we focused on IL-10-producing cells and analyzed the proportion of each immune cell population of interest (Fig. 2H). Even though the frequency of IL-10-producing CD3- cells increased in lung metastasis compared to steady state, the proportion of CD3- cells in IL-10-producing cells reduced. This could be explained by a profound expansion of IL-10-producing Foxp3 + Tregs, from 5.7% in steady state, to 31% in lung metastasis, among all IL-10-producing cells (Fig. 2H). Moreover, Foxp3 + Tregs were indeed the main IL-10-producing cells among all immune cell populations.

Taken together, our results demonstrate that Foxp3 + Tregs are the major cellular source of IL-10 in CRC-derived lung metastasis.

### Foxp3 + Treg-derived IL-10 promotes CRC-derived lung metastasis

**Fig. 3:**
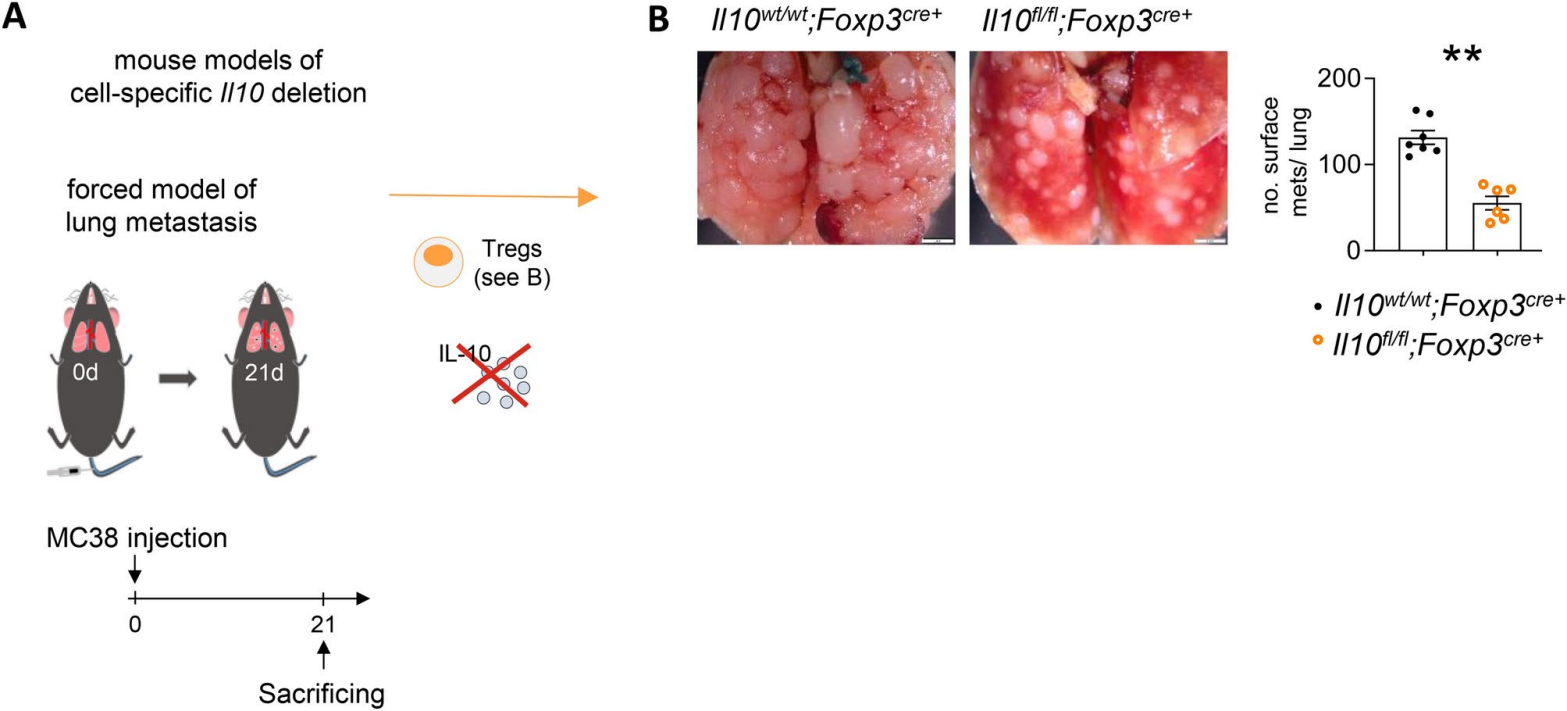
Foxp3 + Tregs-derived IL-10 promotes CRC-derived lung metastasis. (**A**) Schematic overview of lung metastasis induction following MC38 colon cancer cell intravenous injection in mice harboring Foxp3 + Treg cell-specific *Il10* deletion and respective controls (n ≥ 6 mice per group). (**B**) Representative images and number of macroscopic lung metastases. Scale bar: 2 mm. Data are presented as mean ± SEM. **p ≤ 0.01, as calculated by Mann–Whitney *U* test.

Next, we aimed to investigate whether IL-10 expressed by the major producer, Foxp3 + Tregs, could affect lung metastasis development. To this end, we employed another mouse model to delete IL-10 from Foxp3 + Tregs, namely *Il10*^*flox/flox*^*; Foxp3*^*cre*+^ mice*.* Lung metastasis was induced as described before (Fig. 3A), and the lung metastatic load was assessed three weeks later. Indeed, IL-10 deletion in Foxp3 + Tregs resulted in decreased lung metastases (Fig. 3B), a finding suggesting that Foxp3 + Treg-derived IL-10 promotes CRC-derived lung metastasis.

### Foxp3+ Tregs and myeloid cells express high IL-10Ra levels

**Fig. 4:**
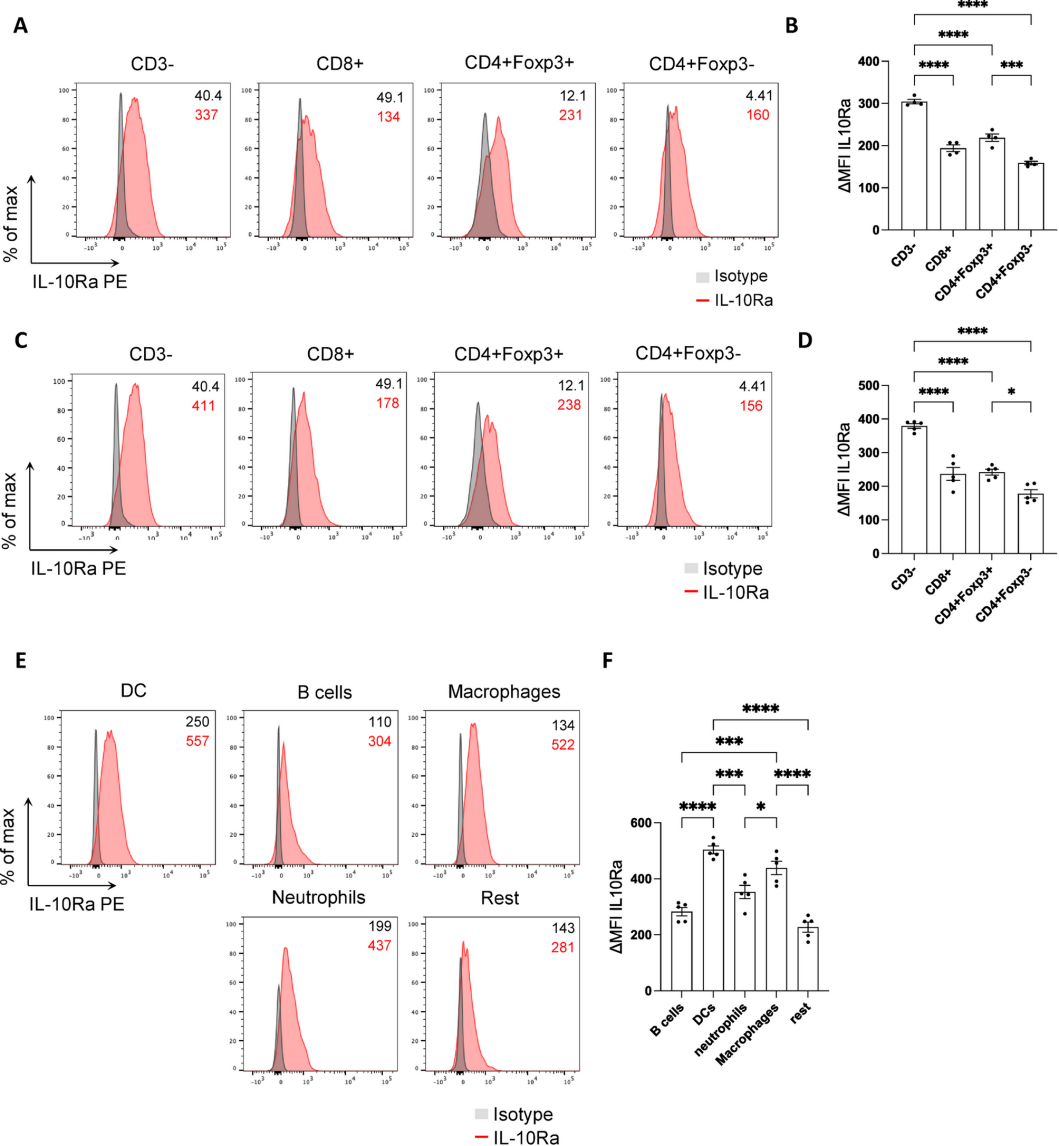
Foxp3 + Tregs and myeloid cells exhibit high IL-10Ra expression. (**A–D**) Representative FACS plots and ΔMFI quantification of IL-10Ra expression in immune cells isolated from (**A**,**B**) healthy lungs (n ≥ 4 mice per group) or (**C**,**D**) lungs with metastasis 21 days post i.v. MC38 cancer cell injection (n ≥ 5 mice per group). (E) Representative FACS plots and (**F**) ΔMFI quantification of IL-10Ra expression in lung CD3- immune cells isolated from lungs with metastasis. Data are presented as mean ± SEM. Non-significant (ns): p > 0.05; *p < 0.05; ***p < 0.001; ****p < 0.0001, as calculated by one-way ANOVA (Bonferroni) with Bonferroni post hoc tests.

As a next step, we aimed to identify the target cells of IL-10 during CRC-derived lung metastasis formation. To this end, we first characterized the IL-10Ra expression in various immune cell populations in the lung. In the healthy lung, CD3- cells exhibited the highest IL-10Ra expression, significantly higher than T cells (Fig. 4A,B). Among T cells, the highest IL-10Ra expression was observed in Foxp3 + Tregs (Fig. 4A,B). Additionally, we determined the IL-10Ra expression in different immune cell populations in lungs with metastasis (Fig. 4C,D). Similar to our findings in steady state conditions, the highest IL-10Ra expression was also identified in CD3- cells in the metastatic lung. Foxp3 + Tregs still exhibited higher IL-10Ra expression compared to other T cell subtypes. Within CD3- cells, dendritic cells (DCs) and macrophages showed higher IL-10Ra levels compared to other immune cell populations (Fig. 4E,F). In conclusion, myeloid cells, especially DCs and macrophages, as well as Foxp3 + Tregs, were found to express high IL-10Ra levels during lung metastasis formation.

### IL-10 signaling in Foxp3+ Tregs and myeloid cells promotes lung metastasis

**Fig. 5:**
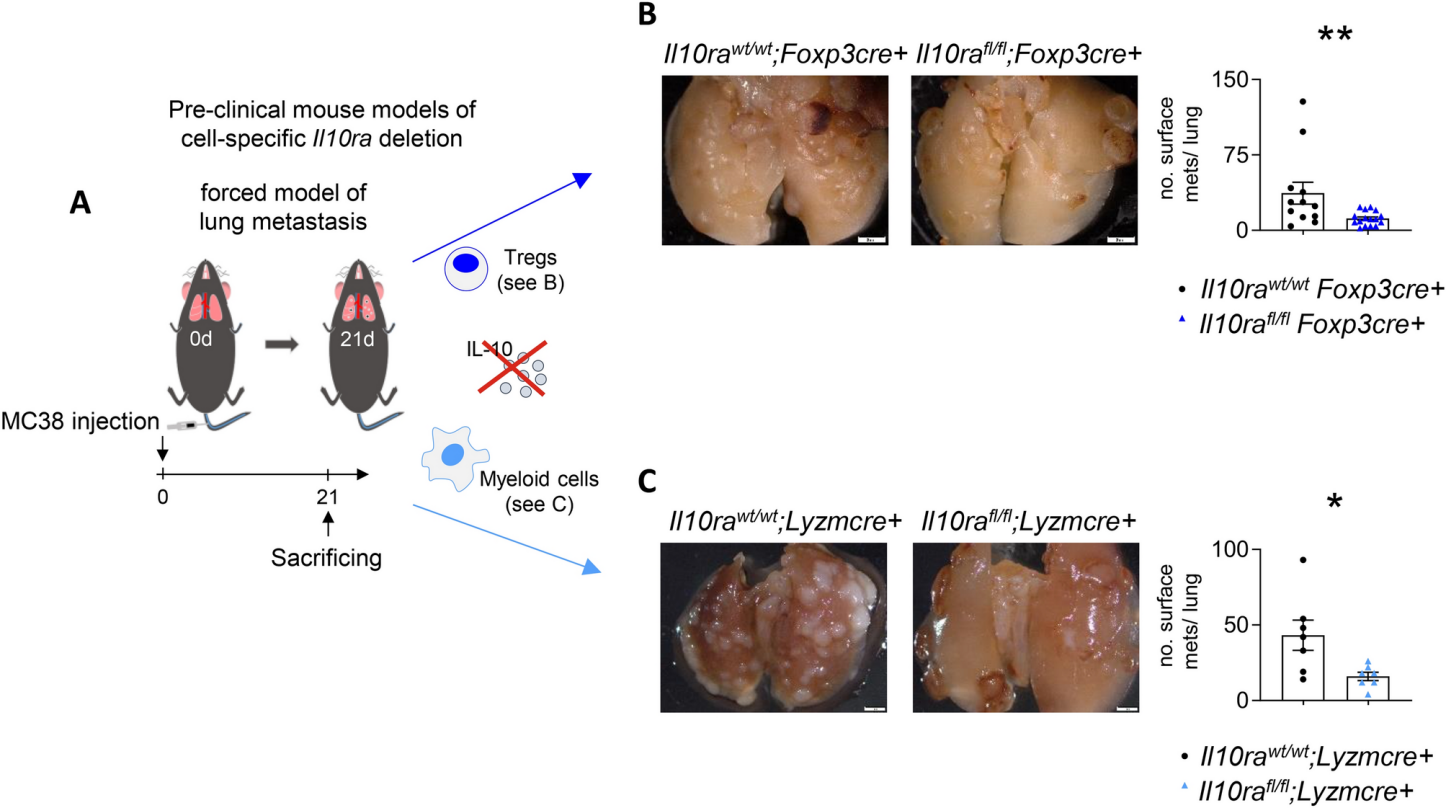
IL-10 signaling in Foxp3 + Tregs and myeloid cells promotes lung metastasis. (**A**) Schematic overview of forced lung metastasis induction in mice with cell-specific *Il10ra* deletion (n ≥ 6 mice per group). (**B**,**C**) Representative images and number of lung metastases in mice with (**B**) Treg−, or (**C**) myeloid cell-specific *Il10ra* deletion. Scale bar: 2 mm. Data are presented as mean ± SEM. Non-significant (ns): p > 0.05; *p < 0.05; **p ≤ 0.01; as calculated by Mann–Whitney *U* test.

Finally, we aimed to examine whether Foxp3 + Tregs and myeloid cells are directly targeted by IL-10 during lung metastasis development. To this end, lung metastasis was induced in transgenic mice allowing a respective cell-specific IL-10Ra deletion in Foxp3 + Tregs (*Il10ra*^*flox/flox*^*; Foxp3*^*cre*+^ mice) or in myeloid cells (*Il10ra*^*flox/flox*^*; Lysm*^*cre*+^ mice).The lungs of these mice were harvested after three weeks (Fig. 5A). Of note, impaired IL-10 signaling in either Foxp3 + Tregs or myeloid cells resulted in lung metastasis reduction (Fig. 5B,C). These findings suggest that Foxp3 + Tregs and myeloid cells not only express high IL-10Ra levels, but also directly mediate the role of IL-10 in lung metastasis formation.

## Discussion

In this study, we investigated the role of IL-10 in lung metastasis. By using the *Il10-*deficient mouse model, we found that, similar to liver metastasis^9^, IL-10 promotes lung metastasis formation. These results highlight the important role of Foxp3 + Treg-derived IL-10 in promoting CRC-derived distant metastasis, through its IL-10Ra-mediated action on myeloid cells and Foxp3 + Tregs. These observations are consistent with previous studies identifying IL-10 as an immunosuppressive agent that can promote tumor growth by inhibiting effective anti-tumor immune responses^22^. Our findings provide a new dimension in understanding the immunological metastatic niche, where IL-10 acts as a facilitator of metastatic progression in both the lung and the liver environment.

Using reporter mice and forced lung metastasis induction, we found that, among all examined immune cell populations, Foxp3 + Tregs are the predominant cellular source of IL-10 in lung metastasis. This is an interesting finding, since reports have often highlighted diverse sources of IL-10 in various diseases, including myeloid-derived suppressor cells (MDSCs) and macrophages^13,23–25^. Here, we demonstrate that Foxp3 + Tregs present as a major IL-10 producing cell source in lung metastasis, similar to liver metastasis, but also indeed functionally contribute to lung metastasis formation. The significant involvement of Foxp3 + Treg-derived IL-10 in metastasis formation suggests that these cells are consistent central drivers for CRC cancer cell colonization and growth in the lung and liver metastatic microenvironment. Of note, lung and liver are the most frequently metastasized organs in CRC patients. This suggests that targeting Foxp3 + Tregs or their IL-10 production may provide a strategic approach for therapeutic interventions aimed at reducing distant metastasis of CRC patients. Higher IL-10 production by CD3- immune cells and CD4 + Foxp3 + Tregs was observed in lungs with metastasis compared to healthy lungs. However, the proportion of CD3- cells in all IL-10-producing cells decreased in lung metastasis, which could be explained by a drastic increase in Foxp3 + Tregs. The fraction of CD4 + Foxp3- cells in IL-10 producing cells was slightly elevated. Although CD4 + Foxp3- cells were not identified as the most predominant IL-10-producing cells, further studies focusing on this particular cell population are warranted.

Furthermore, the higher expression of IL-10Ra on myeloid cells and Foxp3 + Tregs in the metastatic lung was consistent with that in liver metastasis. We have observed that in metastatic lungs, IL-10RA expression was not elevated in T cells, which is similar to our observation in the liver^9^. Nevertheless, we found that IL-10 signaling in T cells does play a role. Thus, it seems that an upregulation of IL-10RA is not essential in order to allow IL-10 to exert its function in metastasis. The fact that deletion of IL-10Ra on myeloid cells and Foxp3 + Tregs resulted in reduced lung metastatic burden, strongly supports a direct effect of IL-10 on these cells during lung metastasis formation. These findings align with our recent reports suggesting that IL-10 signaling in Foxp3 + Tregs and monocytes can facilitate a pro-metastatic microenvironment in the liver^9^.

However, our study has a few limitations. The models used may not fully recapitulate the complexity of human CRC or the heterogeneity of the tumor microenvironment. For example, the microenvironment in lung metastasis may differ upon synchronous presence of liver metastasis, as compared to the sole occurrence of lung metastasis, since the presence of liver metastasis induces systemic immune alterations^26^. However, inducing both lung and liver metastasis in a mouse at the same time will highly burden it, and is therefore not recommended. Future studies could explore the implications of IL-10 signaling in more clinically relevant models, using for example, direct clinical sample-derived organotypic slides. Additionally, therapeutical targeting of IL-10 or its pathway could carry risks given its broad role in controlling inflammation and maintaining immune homeostasis^10,11,13^. A thorough investigation of the side effects of such targeting in clinical settings is warranted^27^.

We recently deciphered the molecular mechanism by which IL-10 promotes metastasis in the liver. IL-10 does not affect cancer cell extravasation, but rather influences later stages of the metastatic cascade. Specifically, Foxp3 + Treg-derived IL-10 acts on Foxp3 + Tregs themselves, thereby amplifying IL-10 production. Furthermore, IL-10 promotes PD-L1 upregulation on monocytes, which subsequently suppresses CD8 + T-cell-mediated immune surveillance and this causing metastasis^9^. Previous studies have shown that deletion of IL-10 or its receptor IL-10Ra can significantly affect the cytotoxic response, particularly involving CD8 + T cells and other cytotoxic effector mechanisms. IL-10 is known for its immunosuppressive role, and in its absence, CD8 + T cell activation typically increases. This is because IL-10 suppresses the production of pro-inflammatory cytokines and costimulatory molecules that are essential for CD8 + T cell activation. CD8 + T cells are more likely to produce higher levels of interferon-gamma (IFN-γ) in the absence of IL-10 or IL-10Ra. IFN-γ is a key cytokine for cytotoxic function and the immune response against tumors. In the absence of IL-10 signaling, CD8 + T cells tend to have higher expression of these molecules, allowing them to more effectively induce apoptosis in target cells^28,29^. Further studies are warranted to test if this mechanism would also apply in lung metastasis.

In conclusion, our data provide evidence that Foxp3 + Tregs are the major cellular source of IL-10 in lung metastasis. Additionally, we demonstrate that Foxp3 + Treg-produced IL-10 promotes CRC-derived lung metastasis formation. Lastly, we report that Foxp3 + Tregs and myeloid cells are direct target cells of IL-10 during lung metastasis formation. These findings highlight the potential therapeutic benefit of IL-10Ra inhibition against CRC-derived lung metastasis. Of note, this study further suggests that anti-IL-10Ra administration could serve as a promising treatment to CRC patients with distant metastasis, without triggering metastatic progression in other organs.

### Limitation of the study

Due to mouse background only one mouse CRC cell line could be used, a point that could be considered as a limitation of the study.

## Supplementary Information

Supplementary Information.

## Data Availability

All data from this study are provided. Primary data from flow cytometry are available upon reasonable request. Further information and requests for resources and reagents should be directed to Anastasios Giannou (a.giannou@uke.de).

## Abbreviations

MC38: Murine colon cancer cells
Tregs: Foxp3 + regulatory T cells
CRC: Colorectal cancer
Pen/Strep: Penicillin–streptomycin
DC: Dendritic cell
i.v.: Intravenous

## References

[1] Cervantes, A. et al. Metastatic colorectal cancer: ESMO Clinical Practice Guideline for diagnosis, treatment and follow-up. *Ann. Oncol.* **34**(1), 10–32 (2023). 36307056 10.1016/j.annonc.2022.10.003

[2] Bray, F. et al. Global cancer statistics 2022: GLOBOCAN estimates of incidence and mortality worldwide for 36 cancers in 185 countries. *CA Cancer J. Clin.* **74**(3), 229–263 (2024). 38572751 10.3322/caac.21834

[3] Gomez-Cuadrado, L., Tracey, N., Ma, R., Qian, B. & Brunton, V. G. Mouse models of metastasis: Progress and prospects. *Dis. Model. Mech.* **10**(9), 1061–1074 (2017). 28883015 10.1242/dmm.030403 PMC5611969

[4] Jordens, M. S. et al. Prevalence of lung metastases among 19,321 metastatic colorectal cancer patients in eight countries of Europe and Asia. *Curr. Oncol.* **28**(6), 5035–5040 (2021). 34940062 10.3390/curroncol28060423 PMC8700218

[5] Mukherjee, A. G. et al. Role of immune cells and receptors in cancer treatment: An immunotherapeutic approach. *Vaccines* **10**(9), 1493 (2022). 36146572 10.3390/vaccines10091493 PMC9502517

[6] Hiam-Galvez, K. J., Allen, B. M. & Spitzer, M. H. Systemic immunity in cancer. *Nat. Rev. Cancer* **21**(6), 345–359 (2021). 33837297 10.1038/s41568-021-00347-z PMC8034277

[7] Leone, R. D. & Powell, J. D. Metabolism of immune cells in cancer. *Nat. Rev. Cancer* **20**(9), 516–531 (2020). 32632251 10.1038/s41568-020-0273-y PMC8041116

[8] Huppert, L. A. et al. Tissue-specific Tregs in cancer metastasis: Opportunities for precision immunotherapy. *Cell Mol. Immunol.* **19**(1), 33–45 (2022). 34417572 10.1038/s41423-021-00742-4 PMC8752797

[9] Shiri, A. M. et al. IL-10 dampens antitumor immunity and promotes liver metastasis via PD-L1 induction. *J. Hepatol.* **78**, S525 (2023). 10.1016/j.jhep.2023.12.015 PMC10964083 38160941

[10] Bedke, T., Muscate, F., Soukou, S., Gagliani, N. & Huber, S. IL-10-producing T cells and their dual functions. *Semin. Immunol.* **44**, 101335 (2019). 31734129 10.1016/j.smim.2019.101335

[11] Rallis, K. S. et al. IL-10 in cancer: An essential thermostatic regulator between homeostatic immunity and inflammation: A comprehensive review. *Future Oncol.* **18**(29), 3349–3365 (2022). 36172856 10.2217/fon-2022-0063

[12] Yogev, N. et al. CD4(+) T-cell-derived IL-10 promotes CNS inflammation in mice by sustaining effector T cell survival. *Cell Rep.* **38**(13), 110565 (2022). 35354043 10.1016/j.celrep.2022.110565

[13] Mannino, M. H. et al. The paradoxical role of IL-10 in immunity and cancer. *Cancer Lett.* **367**(2), 103–107 (2015). 26188281 10.1016/j.canlet.2015.07.009

[14] Saraiva, M., Vieira, P. & O’Garra, A. Biology and therapeutic potential of interleukin-10. *J. Exp. Med.* **217**(1), 418 (2020). 10.1084/jem.20190418 PMC7037253 31611251

[15] Shouval, D. S. et al. Interleukin 10 receptor signaling: Master regulator of intestinal mucosal homeostasis in mice and humans. *Adv. Immunol.* **122**, 177–210 (2014). 24507158 10.1016/B978-0-12-800267-4.00005-5 PMC4741283

[16] Silva, F. S. et al. A dual-role for IL-10: From leukemogenesis to the tumor progression in acute lymphoblastic leukemia. *Cytokine* **171**, 156371 (2023). 37725872 10.1016/j.cyto.2023.156371

[17] Obenauf, A. C. & Massague, J. Surviving at a distance: Organ-specific metastasis. *Trends Cancer* **1**(1), 76–91 (2015). 28741564 10.1016/j.trecan.2015.07.009 PMC4673677

[18] Izraely, S. & Witz, I. P. Site-specific metastasis: A cooperation between cancer cells and the metastatic microenvironment. *Int. J. Cancer* **148**(6), 1308–1322 (2021). 32761606 10.1002/ijc.33247 PMC7891572

[19] Rubtsov, Y. P. et al. Regulatory T cell-derived interleukin-10 limits inflammation at environmental interfaces. *Immunity* **28**(4), 546–558 (2008). 18387831 10.1016/j.immuni.2008.02.017

[20] Lucke, J. et al. Protocol for generating lung and liver metastasis in mice using models that bypass intravasation. *STAR Protoc.* **5**(1), 102696 (2024). 38244200 10.1016/j.xpro.2023.102696 PMC10831314

[21] Kamanaka, M. et al. Expression of interleukin-10 in intestinal lymphocytes detected by an interleukin-10 reporter knockin tiger mouse. *Immunity* **25**(6), 941–952 (2006). 17137799 10.1016/j.immuni.2006.09.013

[22] Sullivan, K. M. et al. Blockade of interleukin 10 potentiates antitumour immune function in human colorectal cancer liver metastases. *Gut* **72**, 325–337 (2022). 35705369 10.1136/gutjnl-2021-325808 PMC9872249

[23] Yaseen, M. M., Abuharfeil, N. M., Darmani, H. & Daoud, A. Mechanisms of immune suppression by myeloid-derived suppressor cells: The role of interleukin-10 as a key immunoregulatory cytokine. *Open Biol.* **10**(9), 200111 (2020). 32931721 10.1098/rsob.200111 PMC7536076

[24] Shouval, D. S. et al. Interleukin-10 receptor signaling in innate immune cells regulates mucosal immune tolerance and anti-inflammatory macrophage function. *Immunity* **40**(5), 706–719 (2014). 24792912 10.1016/j.immuni.2014.03.011 PMC4513358

[25] Mocellin, S., Marincola, F. M. & Young, H. A. Interleukin-10 and the immune response against cancer: A counterpoint. *J. Leukoc. Biol.* **78**(5), 1043–1051 (2005). 16204623 10.1189/jlb.0705358

[26] Yu, J. et al. Liver metastasis restrains immunotherapy efficacy via macrophage-mediated T cell elimination. *Nat. Med.* **27**(1), 152–64 (2021). 33398162 10.1038/s41591-020-1131-x PMC8095049

[27] Ganesh, K. & Massague, J. Targeting metastatic cancer. *Nat. Med.* **27**(1), 34–44 (2021). 33442008 10.1038/s41591-020-01195-4 PMC7895475

[28] Jaime-Sanchez, P. et al. Cell death induced by cytotoxic CD8+ T cells is immunogenic and primes caspase-3-dependent spread immunity against endogenous tumor antigens. *J. Immunother. Cancer* **8**(1), e000528 (2020). 32241808 10.1136/jitc-2020-000528 PMC7174069

[29] Groux, H., Bigler, M., de Vries, J. E. & Roncarolo, M. G. Inhibitory and stimulatory effects of IL-10 on human CD8+ T cells. *J. Immunol.* **160**(7), 3188–3193 (1998). 9531274

