# Author Correction: Foxp3 + Treg-derived IL-10 promotes colorectal cancer-derived lung metastasis

**DOI:** 10.1038/s41598-025-17854-w

**Published:** 2025-09-23

**Authors:** Ahmad Mustafa Shiri, Mohammad Fard-Aghaie, Tanja Bedke, Eleftherios D. Papazoglou, Morsal Sabihi, Dmitra E. Zazara, Siwen Zhang, Jöran Lücke, Philipp Seeger, Maximilian Evers, Thilo Hackert, Karl J. Oldhafer, Gabriel E. Gondolesi, Samuel Huber, Anastasios D. Giannou

**Affiliations:** 1 Section of Molecular Immunology and Gastroenterology, I. Department of Medicine, University Medical Center Hamburg-Eppendorf, 20246 Hamburg, Germany; 2 Hamburg Center for Translational Immunology (HCTI), University Medical Center Hamburg-Eppendorf, 20246 Hamburg, Germany; 3 Department of General, Visceral and Thoracic Surgery, University Medical Center Hamburg-Eppendorf, 20246 Hamburg, Germany; 4 Division for Experimental Feto-Maternal Medicine, Department of Obstetrics and Fetal Medicine, University Medical Center Hamburg-Eppendorf, Hamburg, Germany; 5 University Children’s Hospital, University Medical Center Hamburg-Eppendorf, Hamburg, Germany; 6 Division of Hepatobiliary and Pancreatic Surgery, Department of Surgery, Asklepios Hospital Barmbek, Hamburg, Germany; 7 Semmelweis University Budapest, Asklepios Campus Hamburg, Hamburg, Germany; 8 General Surgery, Liver, Pancreas and Intestinal Transplantat Unit, Hospital Universitario-Fundación Favaloro, Buenos Aires, Argentina; 9 Section of Molecular Immunology and Gastroenterology, I. Department of Medicine, Center of Internal Medicine and Department of General, Visceral and Thoracic Surgery, University Medical Center Hamburg-Eppendorf, 20246 Hamburg, Germany

Correction to: *Scientific Reports* 10.1038/s41598-024-80437-8, published online 16 December 2024

The original version of this Article contained errors.

In the original version of this Article, Ahmad Mustafa Shiri and Mohammad Fard-Aghaie were omitted as equally contributing authors.

In addition, due to an error during figure assembly in Figure 2C, the FACS panel for the condition “steady state” CD3 + shows the same image as the condition “metastasis” CD3-. In addition, the FACS profiles in Figures 2B–E and Figure 4A, C, E are missing numbers on all axes.

The original, incorrect Figures 2 and 4 are shown below as Figures 1 and 2, respectively.

**Fig. 1:**
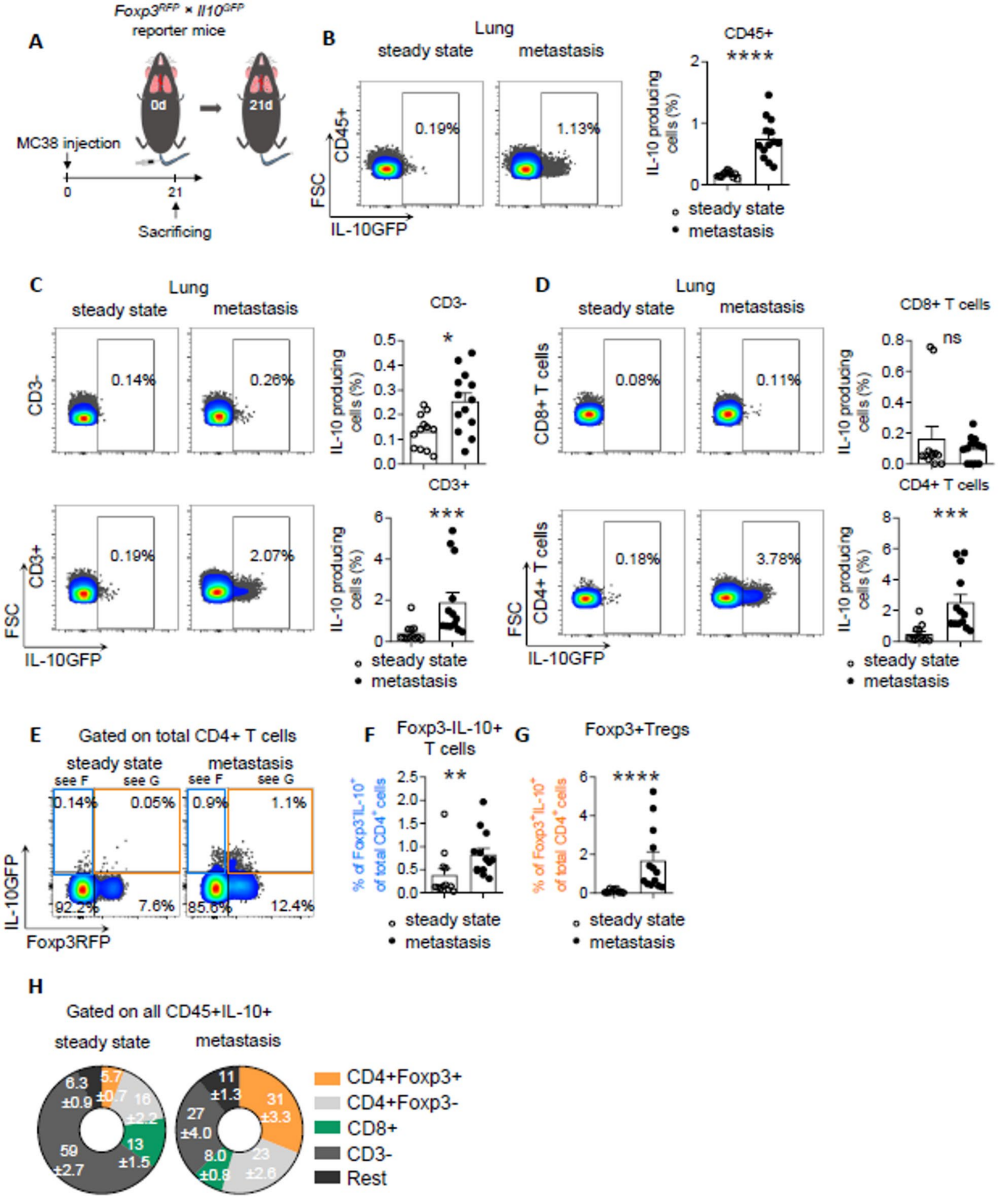
Foxp3 + Tregs are the major source of IL-10 in lung metastasis formation. (**A**) Schematic overview of the forced lung metastasis induction using intravenous injection of MC38 cancer cells in *Foxp3*^*RFP*^; *Il10*^*GFP*^ reporter mice (n ≥ 12 mice per group). (**B**–**D**) Frequency of IL-10 + cells in (**B**) all CD45 + cells, (**C**) CD3- cells and T cells, and in (**D**) CD8 + T cells as well as in CD4 + T cells. (**E**–**G**) IL-10 expression in (**F**) Foxp3- IL-10 + cells and (**G**) Foxp3 + Tregs. (**H**) General distribution of all IL-10 producing CD45 + cells in healthy lung and lung with metastasis. Data are presented as mean ± SEM. Non-significant (ns): p > 0.05; *p < 0.05; **p ≤ 0.01; ***p < 0.001, as calculated by Mann–Whitney *U* test.

**Fig. 2:**
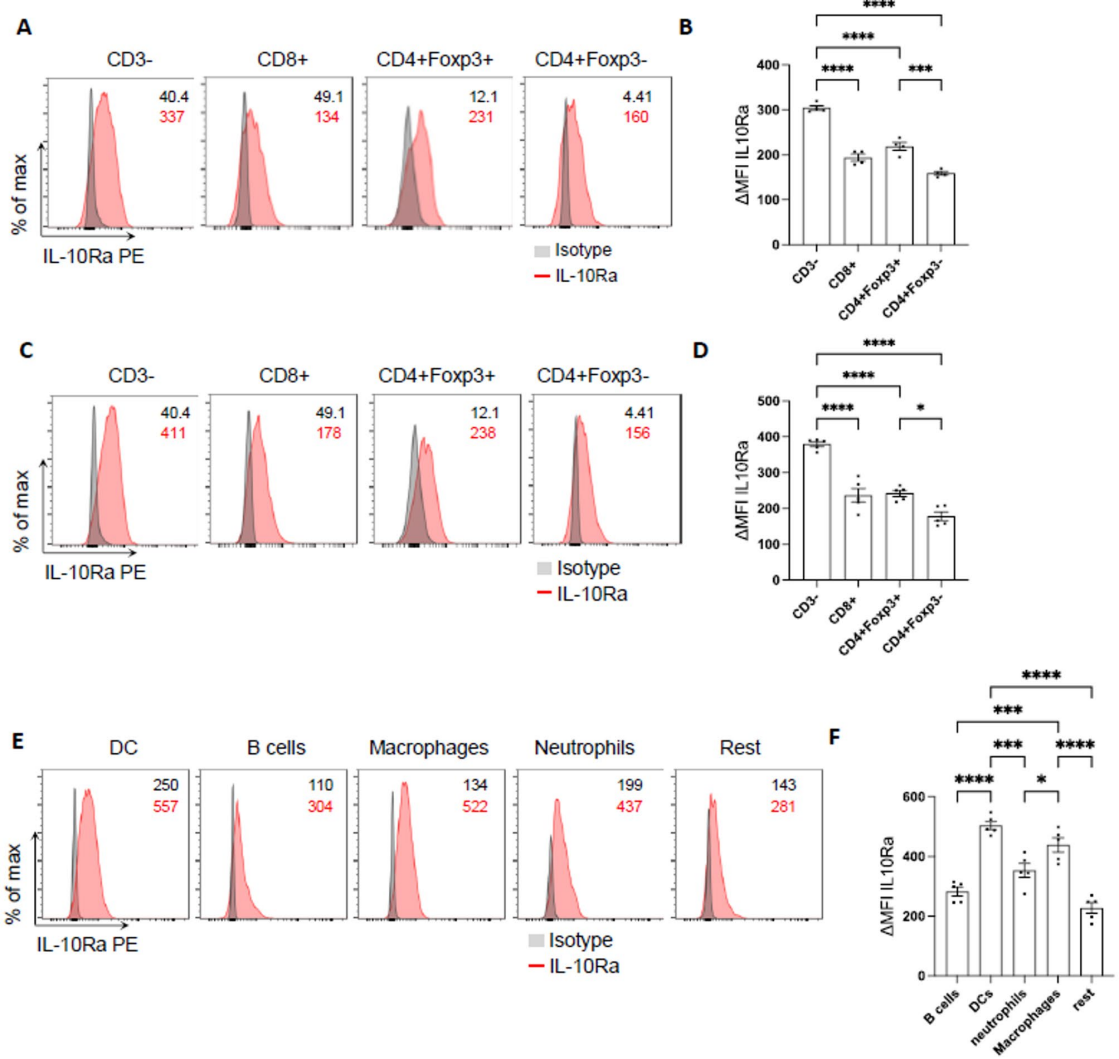
Foxp3 + Tregs and myeloid cells exhibit high IL-10Ra expression. (**A**–**D**) Representative FACS plots and ΔMFI quantification of IL-10Ra expression in immune cells isolated from (**A**, **B**) healthy lungs (n ≥ 4 mice per group) or (**C**, **D**) lungs with metastasis 21 days post i.v. MC38 cancer cell injection (n ≥ 5 mice per group). (E) Representative FACS plots and (**F**) ΔMFI quantification of IL-10Ra expression in lung CD3- immune cells isolated from lungs with metastasis. Data are presented as mean ± SEM. Non-significant (ns): p > 0.05; *p < 0.05; ***p < 0.001; ****p < 0.0001, as calculated by one-way ANOVA (Bonferroni) with Bonferroni post hoc tests.

The original Article has been corrected.

