# Supplementary material for: Foxp3 + Treg-derived IL-10 promotes colorectal cancer-derived lung metastasis
Source: Sci Rep. 2024 Dec 16;14:30483. doi: 10.1038/s41598-024-80437-8 (PMC11649764; doi:10.1038/s41598-024-80437-8)
Supplement: Supplementary file 1 — Supplementary Information. [file 41598_2024_80437_MOESM1_ESM.pdf]

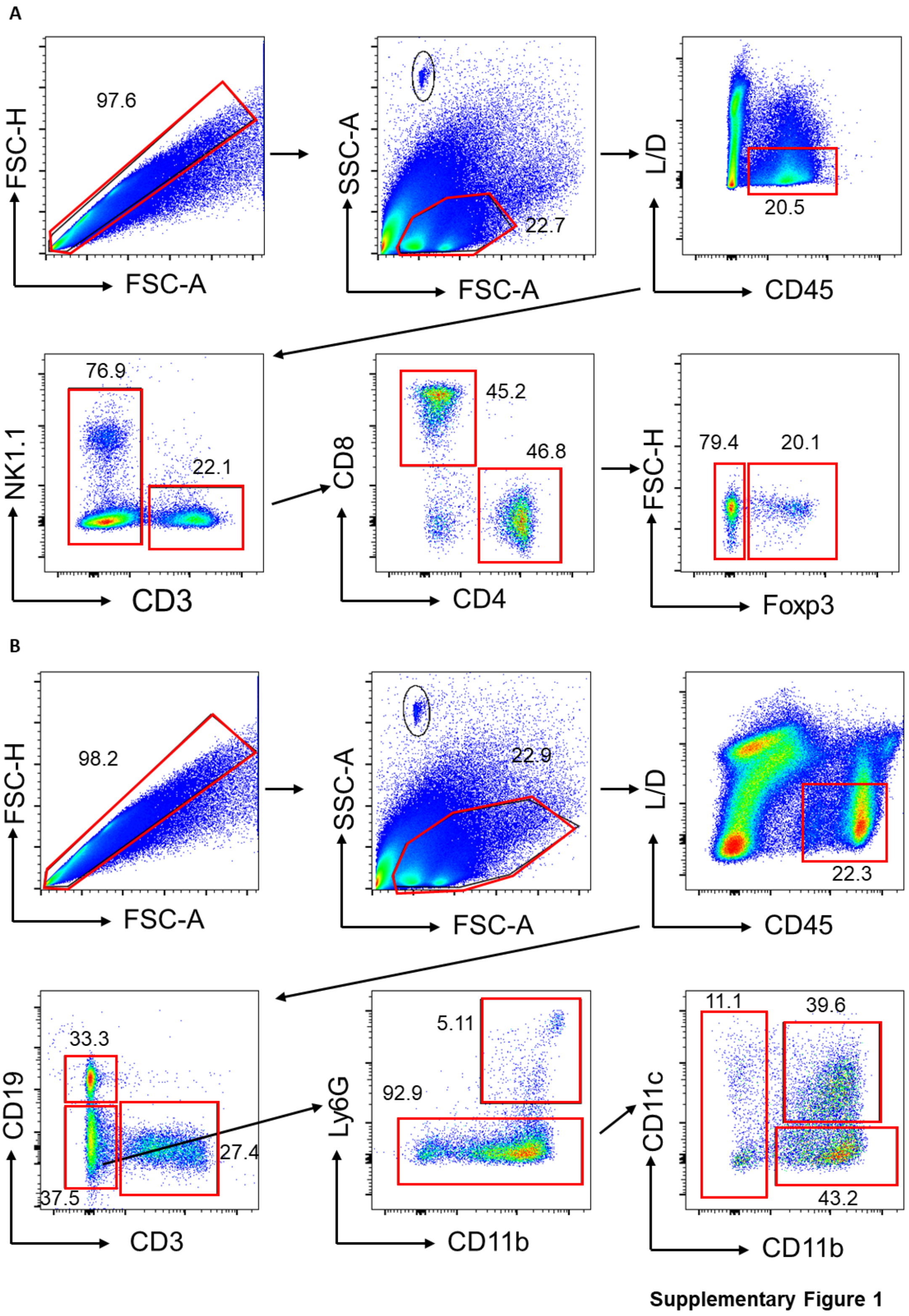

Supplementary Fig.1: Gating strategy for Figure 4

Supplementary Table 1: Flow cytometry antibodies utilized for this study

Name	Supplier	Cat no.	Clone no.
Anti-mouse CD45 BV785	Biolegend	103149	30-F11
Anti-mouse CD45 BUV395	BD	564279	30-F11
Anti-mouse CD3 BV421	Biolegend	100228	17A2
Anti-mouse CD3 PE-Dazzle	Biolegend	100246	17A2
Anti-mouse CD3 BV650	Biolegend	100229	17A2
Anti-mouse CD3 BUV395	BD	740268	17A2
Anti-mouse CD4 APC	Biolegend	100412	GK1.5
Anti-mouse CD4 BUV737	BD	612761	GK1.5
Anti-mouse CD8 PE-Cy7	Biolegend	100722	53-6.7
Anti-mouse IL-10Ra PE	Biolegend	112706	1B1.3a
Isotype PE	Biolegend	400408	RTK2071
Anti-mouse CD11b Pacblue	Biolegend	101224	M1/70
Anti-mouse CD11c APC	Biolegend	117312	N418
Anti-mouse Ly6G AF488	Biolegend	127625	1A8
Anti-mouse Ly6C PE	Biolegend	560592	AL-21
Anti-mouse CD19 APC-Cy7	Biolegend	115530	6D5
Fixable Viability Dye eFluor 506	Invitrogen	65-0866-14	None
